# Retraction Note: TRIM11 overexpression promotes proliferation, migration and invasion of lung cancer cells

**DOI:** 10.1186/s13046-023-02721-1

**Published:** 2023-06-01

**Authors:** Xiaolin Wang, Weiping Shi, Hongcan Shi, Shichun Lu, Kang Wang, Chao Sun, Jiansheng He, Weiguo Jin, Xiaoxia Lv, Hui Zou, Yusheng Shu

**Affiliations:** Department of Thoracic Surgery, Northern Jiangsu People’s Hospital and Clinical Medical College of Yangzhou University Yangzhou, No. 98 Nantong West Road, Yangzhou, 225001 People’s Republic of China

**Retraction Note: *J Exp Clin Cancer Res *35, 100 (2016)** 10.1186/s13046-016-0379-y

The Editor-in-Chief has retracted this article. Concerns were raised regarding a number of figures, specifically:

- Figure 2d: the TRIM11 NCI-H446 band appears to be identical to the PCNA band of Fig. 5C in an article by different authors that was simultaneously under consideration with another journal [1].
- Figure 5a: the p-AKT band appears to be identical to the the STK39 NCI-H358 band of Fig. 3C in an article by different authors that was simultaneously under consideration with another journal [2].

The Editor-in-Chief therefore no longer has confidence in the results and conclusions reported in this article.

Authors Yusheng Shu has not responded to correspondence regarding this retraction. The Publisher has not been able to obtain a current email address for authors Xiaolin Wang, Weiping Shi, Hongcan Shi, Shichun Lu, Kang Wang, Chao Sun, Jiansheng He, Weiguo Jin, Xiaoxia Lv and Hui Zou.
